# Effect of Erythropoiesis-Stimulating Agents on Blood Pressure in Pre-Dialysis Patients

**DOI:** 10.1371/journal.pone.0084848

**Published:** 2013-12-31

**Authors:** Marit M. Suttorp, Tiny Hoekstra, Moshe Mittelman, Ilka Ott, Casper F. M. Franssen, Friedo W. Dekker

**Affiliations:** 1 Department of Clinical Epidemiology, Leiden University Medical Center, Leiden, The Netherlands; 2 Department of Medicine, Tel Aviv Sourasky Medical Center, Sackler Faculty of Medicine, Tel Aviv, Israel; 3 Deutsches Herzzentrum der Technischen Universität München, München, Germany; 4 Department of Nephrology, University Medical Center Groningen, Groningen, The Netherlands; Baker IDI Heart and Diabetes Institute, Australia

## Abstract

**Introduction:**

Erythropoiesis-Stimulating Agents (ESA) are hypothesized to increase cardiovascular mortality in patients with chronic kidney disease. One of the proposed mechanisms is the elevation of blood pressure (BP) by ESA. Therefore, we aimed to determine whether the use of ESA was associated with antihypertensive treatment and higher BP.

**Materials and Methods:**

In this cohort 502 incident pre-dialysis patients were included who started specialized pre-dialysis care in 25 clinics in the Netherlands. Data on medication including ESA use and dose, co-morbidities and BP were routinely collected every 6 months. Antihypertensive treatment and BP were compared for patients with and without ESA at baseline. Differences in antihypertensive medication and BP during pre-dialysis care were estimated with linear mixed models adjusted for age, sex, body mass index, cardiovascular disease, diabetes mellitus and estimated glomerular filtration rate.

**Results:**

At baseline, 95.6% of patients with ESA were treated with antihypertensive medication and 73.1% of patients without ESA. No relevant difference in BP was found. During pre-dialysis care patients with ESA used 0.77 (95% CI 0.63;0.91) more classes of antihypertensive drugs. The adjusted difference in systolic blood pressure (SBP) was −0.3 (95% CI −2.7;2.0) mmHg and in diastolic blood pressure (DBP) was −1.0 (95% CI −2.1;0.3) mmHg for patients with ESA compared to patients without ESA. Adjusted SBP was 3.7 (95% CI −1.6;9.0) mmHg higher in patients with a high ESA dose compared to patients with a low ESA dose.

**Conclusions:**

Our study confirms the hypertensive effect of ESA, since ESA treated patients received more antihypertensive agents. However, no relevant difference in BP was found between patients with and without ESA, thus the increase in BP seems to be controlled for by antihypertensive medication.

## Introduction

Hypertension is present in 71–95% of adult patients with chronic kidney disease (CKD) [1]–[4]. High blood pressure (BP) is a major risk factor for cardiovascular morbidity and mortality [5] and is associated with an increased loss of renal function [6]. Therefore guidelines recommend a BP control to less than 130/80 mmHg for patients with CKD [7]–[9].

With declining renal function, endogenous erythropoietin production decreases and the majority of CKD patients will be treated with erythropoiesis-stimulating agents (ESAs) for their anemia. However, recently several anemia-correction trials in pre-dialysis patients have demonstrated that patients randomized to achieve normal hemoglobin levels experience more cardiovascular events or a higher mortality rate [10]–[12]. These patients were treated with higher ESA doses than patients assigned to the lower hemoglobin arm. Some observational studies have also shown an increased all-cause and cardiovascular mortality in dialysis patients treated with higher ESA doses [13], [14]. The mechanism responsible for these excess cardiovascular events and mortality is not entirely clarified. Several hypotheses have been proposed, but the elevation of BP by ESAs thereby increasing the risk of cardiovascular events is one of the most important theories [15], [16].

Indeed ESA induced hypertension has already been reported at the introduction of ESAs late 1980s in 10–32% of hemodialysis patients [17]–[19]. However, since then nephrologists have learned to slowly increase hemoglobin with lower ESA doses to avoid these side effects. Prior studies have also not consistently identified differences in BP in patients randomized to higher versus lower hemoglobin targets [11], [12], [20], [21]. In clinical practice actual BP can remain stable during ESA treatment, with or without adjustments in antihypertensive medication [19], [22].

Data on BP control is limited in patients with CKD and the available studies underrepresent the pre-dialysis patients [2]–[4], [23]–[25]. Most importantly, information about the influence of ESA therapy and especially high doses of ESA therapy on BP is lacking. Therefore we aimed to determine whether the use of ESA was associated with antihypertensive treatment and higher BP.

## Materials and Methods

### Study Design and Population

The PREdialysis PAtient REcord (PREPARE-2) study is a prospective follow-up study of incident pre-dialysis patients treated in 25 nephrology outpatient clinics in the Netherlands. Patients of at least eighteen years of age were included at the start of specialized pre-dialysis care between July 2004 and June 2011. In practice, this refers to incident pre-dialysis patients with an estimated glomerular filtration rate (eGFR) of less than 20–30 mL/min/1.73 m^2^, in whom renal function loss is progressive. Patients with a failing kidney transplant were also included in the study if the transplantation was at least one year ago. All participants gave their written informed consent prior to study inclusion. The patients were treated by their nephrologists in their regular scheme according to the treatment guideline of the Dutch Federation of Nephrology [26], a Dutch guideline based on the KDOQI guidelines [7], [27]. Clinical data were collected at the start of specialized pre-dialysis care and in subsequent 6-month intervals. Patients were followed until the start of dialysis, transplantation, death, or censoring. Censoring was defined as moving to an outpatient clinic not participating in the PREPARE-2 study, recovery of renal function, refusal of further study participation, lost to follow-up or reaching the end of follow-up at August 1, 2012, whichever came first. The study was reviewed and approved by the medical ethics committee of the Leiden University Medical Center. The medical ethics committee or institutional review board (as appropriate) of all participating centers additionally reviewed and approved the local feasibility of the study (see Supporting Information File S1).

### Measurements and Definitions

Data on demography, primary kidney disease, co-morbidities and medication use were collected at the start of specialized pre-dialysis care and in subsequent 6-month intervals by the patients’ nephrologist or specialized nurse. Corresponding laboratory data were extracted from the electronic hospital information systems or medical records. Body mass index (BMI) was calculated as weight (kg) divided by height (m) squared. Primary kidney disease was classified according to the codes of the European Renal Association-European Dialysis and Transplantation Association and grouped into four categories (diabetes mellitus, glomerulonephritis, renal vascular disease and other) [28]. eGFR was calculated using the abbreviated MDRD-formula, taking sex, age, race and measured serum creatinine into account [29]. ESA dose was registered in units per week, for darbepoetin dose in micrograms was converted to units by multiplying with 200. ESA dose was categorized in four subsequent dosing intervals: ≤2000 units/week, 2001–4000 units/week, 4001–6000 units/week and >6000 units/week.

### Outcome

Antihypertensive drugs were grouped into 8 classes (beta blockers, calcium channel blockers, ACE inhibitors, angiotensin receptor blockers, loop diuretics, other diuretics, alpha blockers and others). Combination drugs were described in terms of their components and then the number of antihypertensive drug classes was counted. BP was measured as part of usual care by nephrologists or clinical nurses in each outpatient clinic. In the Netherlands, the standardized procedure for measuring BP is with the use of cuff occlusion of the arm and auscultation when the patient is in sitting position after five minutes of rest. With an appropriate sized cuff at the height of the heart, at least two measurements one to two minutes apart are performed. More measurements are performed when the first two measurements are clearly different and the mean of (the last) two values is noted [30]. Within BP targets was defined as a systolic blood pressure (SBP) <130 mmHg and diastolic blood pressure (DBP) <80 mmHg, as recommended in guidelines [7]–[9].

### Statistical Analyses

Baseline characteristics were presented for the total study population and stratified for patients with and without ESA. Continuous data were expressed as mean (standard deviation) and categorical data as percentages. BP and antihypertensive medication was compared for patients with and without ESA at baseline with an unpaired Student’s t-test or chi-square test. The effect of ESA use and dose on the number of antihypertensive drug classes or BP during pre-dialysis care was estimated using linear mixed effects models. The models were used as repeated cross-sectional analyses to estimate the difference in antihypertensive medication, SBP and DBP between patients with and without ESA treatment. To estimate the effect of ESA dose on BP, the difference in SBP and DBP was also estimated in patients with ESA treatment in subsequent dose intervals. To account for correlation between measurements within the same patient a random intercept for patients was applied. The models were checked for interaction between time and ESA use or dose and eGFR and ESA use and dose. The analyses were adjusted for age, sex, BMI, diabetes mellitus, cardiovascular disease and eGFR. Of all BP measurements at different time points during pre-dialysis care, corresponding BMI was missing in 3.8% and eGFR in 25.7%. Missing data on BMI and eGFR were imputed with standard multiple imputation techniques in SPSS with 20 imputation sets, which are based on the Markov Chain Monte Carlo (MCMC) method [31].

### Sensitivity Analyses

To further quantify the intensity of antihypertensive drug treatment, a standardized daily dose was calculated by dividing the daily prescribed milligrams of drug by the drug’s defined daily dose (DDD). DDD is the average daily dose of a drug taken by adults for its main indication, developed by the World Health Organization for use in drug utilization studies [32]. To obtain a total standardized daily dose, all antihypertensive drug specific standardized doses were added up, reflecting both the total number and total dose of antihypertensive medication use. To compare total standardized daily doses between patients with and without ESA, a linear mixed model was used as described in the previous section. Furthermore, a sensitivity analysis excluding patients with a renal transplant was performed. The excluded renal transplant patients were identified by their use of immunosuppressive medication.

All statistical analyses were performed with SPSS statistical software, version 20 (IBM Corp, Armonk NY).

## Results

### Demographic and Clinical Characteristics

A total of 502 patients were included in the study, of which 205 (40.8%) patients were treated with ESA at the start of pre-dialysis care. A summary of demographic and clinical characteristics at baseline is shown in Table 1. Mean age was 64.9 years, 67.9% was male and mean eGFR was 16.6 ml/min/1.73 m^2^. In ESA treated patients mean eGFR was somewhat lower than in patients without ESA treatment.

**Table 1:** Characteristics of patients with and without ESA at the start of pre-dialysis care.

	all patients	No ESA	ESA
Number	502	297	205
Age	64.9 (14.3)	64.4 (14.5)	65.7 (14.1)
Sex (% male)	67.9	67.7	68.3
BMI (kg/m2)	26.8 (5.2)	26.6 (5.3)	27.0 (5.0)
Primary kidney disease (%)			
Diabetes mellitus	14.3	13.5	15.6
Glomerulonefritis	13.3	12.8	14.1
Renal vascular disease	30.7	30.3	31.2
Other	41.6	43.4	39.0
Comorbidity (%)			
Diabetes Mellitus	26.3	24.2	29.3
Hypertension	83.2	84.8	80.9
Cardiovascular disease	41.2	43.4	38.0
eGFR (MDRD) mL/min/1.73 m2	16.6 (5.9)	17.4 (5.8)	15.6 (6.0)
Albumin (g/L)	40.7 (4.6)	41.0 (4.7)	40.4 (4.4)
Hemoglobin (g/dL)	12.3 (1.5)	12.4 (1.5)	12.1 (1.4)

Values are presented as mean (standard deviation) or percentage.

Abbreviations: ESA = Erythropoiesis-Stimulating Agent, BMI = Body Mass Index, eGFR = estimated Glomerular Filtration Rate.

### Hypertension Treatment and BP at Baseline

**Figure 1:**
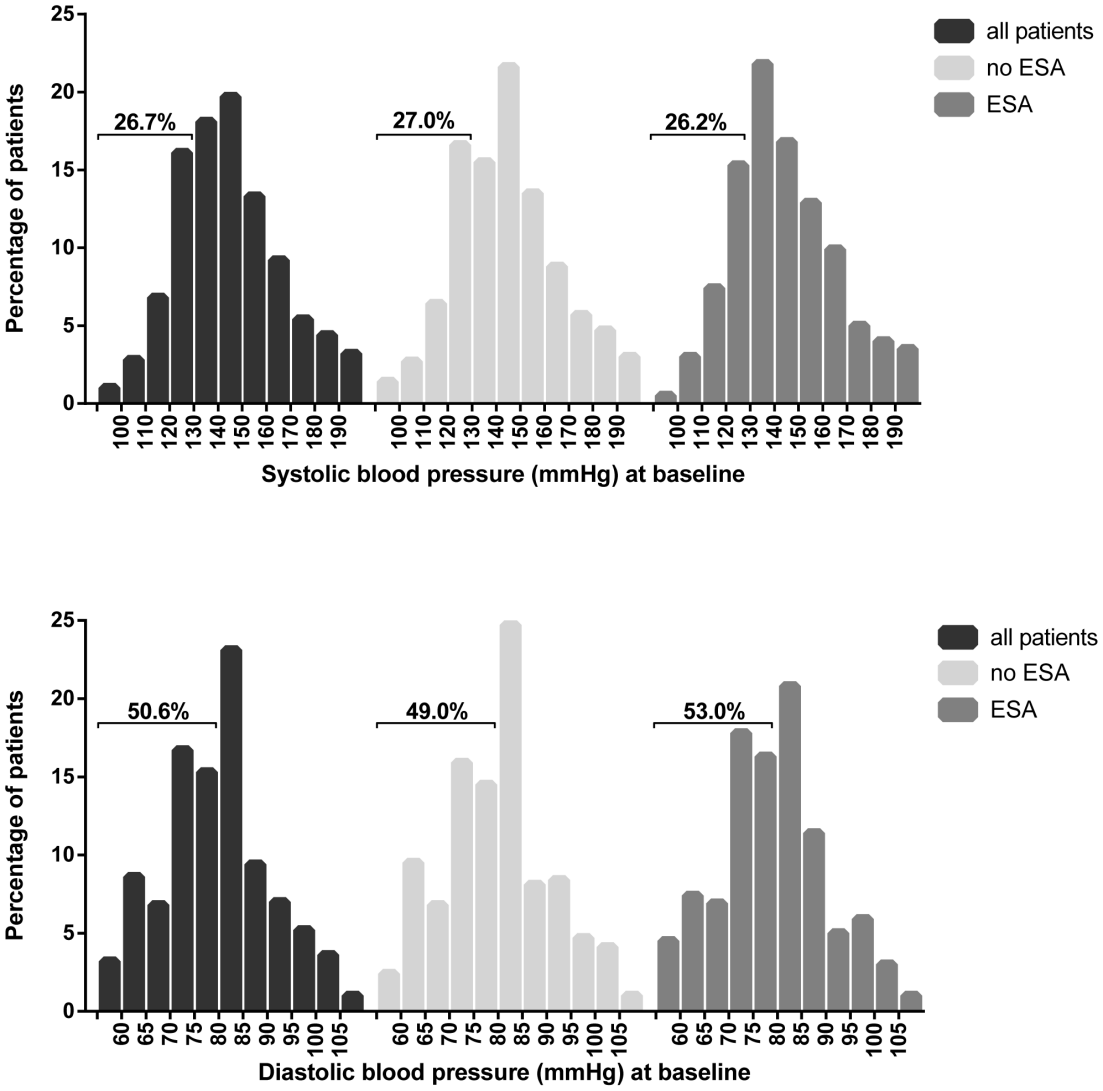
Distribution of systolic and diastolic blood pressure at baseline. The distribution of SBP and DBP are presented for the total population and stratified by ESA use. The total percentage of patients within the blood pressure target (DBP: 80 mmHg and SBP: 130 mmHg) are depicted in the histogram. Abbreviations: ESA = Erythropoiesis-Stimulating Agent, SBP = Systolic Blood Pressure, DBP = Diastolic Blood Pressure.

Antihypertensive medication was prescribed in 95.6% of ESA treated patients as opposed to 73.1% of patients without ESA at the start of pre-dialysis care (Table 2), and the number of antihypertensive drug classes was generally higher. Mainly angiotensin receptor blockers and loop diuretics were prescribed more often in ESA treated patients. Distributions of SBP and DBP at baseline are shown in Figure 1. Overall mean SBP was 142 mmHg and DBP was 78 mmHg, there was no difference in patients with and without ESA. The percentage of patients within BP targets (SBP≤130 mmHg and DBP≤80 mmHg) was the same in patients with and without ESA. SBP was adequately controlled in 26.7% of all patients, DBP in 50.6% and both in 20.3%.

**Table 2:** Blood pressure and antihypertensive drugs in patients with and without ESA at the start of pre-dialysis care.

	all patients	No ESA	ESA	p
Number	502	297	205	
Treated with antihypertensive drugs (%)	82.3	73.1	95.6	<0.01
Number of antihypertensive drug classes (%)				
0	17.7	26.9	4.4	<0.01
1	11.6	8.8	15.6	
2	21.9	20.2	24.4	
3	23.5	20.9	27.3	
4	16.9	15.8	18.5	
5	6.6	5.7	7.8	
6	1.8	1.7	2.0	
Classes of drugs (%)				
Beta blockers	46.0	45.5	46.8	0.76
Calcium channel blockers	46.6	43.1	51.7	0.06
ACE inhibitors	42.0	40.1	44.9	0.28
Angiotensin receptor blockers	37.5	31.0	46.8	<0.01
Loop diuretics	35.5	27.3	47.3	<0.01
Other diuretics	19.7	19.2	20.5	0.72
Alpha blockers	8.0	5.7	11.2	0.03
Others	1.8	2.0	1.5	0.64
SBP	142 (22)	143 (23)	142 (21)	0.86
DBP	78 (12)	78 (12)	78 (11)	0.68
Within BP targets (%)	20.3	20.6	19.8	0.83

Values are presented as mean (standard deviation) or percentage. Differences between patients with and without ESA were tested with a t-test or chi square test, as appropriate.

Abbreviations: ESA = Erythropoiesis-Stimulating Agent, ACE = Angiotensin Converting Enzyme, SBP = Systolic Blood Pressure, DBP = Diastolic Blood Pressure, BP = Blood Pressure.

### ESA use and Antihypertensive Medication during Pre-dialysis Care

The percentage of patients using ESA increased from 41% to more than 50% within the first years of pre-dialysis care. Of the patients that started dialysis, 58.3% was treated with ESA at their last regular measurement before the start of dialysis. The percentage of patients using antihypertensive medication increased over time from 95.5% to 100% in ESA using patients and from 73.0% to 100% in patients without ESA.

### Antihypertensive Medication during Pre-dialysis Care

Mean number of antihypertensive drug classes and BP in patients with and without ESA treatment is shown in Table 3. Patients with ESA treatment used more antihypertensive drugs to control their BP, with an average difference of 0.77 drug classes (95% confidence interval (CI) 0.63;0.91). This means that at least three out of four patients with ESA were treated with one antihypertensive class more than patients without ESA. Sensitivity analysis with total standardized daily dose confirmed the increased antihypertensive drug use: patients with ESA treatment were treated with 1.61 (95% CI 1.12;2.10) standardized daily doses of antihypertensive drugs more than patients without ESA treatment.

**Table 3:** Difference in antihypertensive drugs and BP in patients with and without ESA.

	ESA	Unadjusted		Adjusted^1^		Adjusted^2^	
**Number of antihypertensive**	No	ref		ref		ref	
**drug classes**	Yes	0.79	(0.64;0.93)	0.78	(0.64;0.92)	0.77	(0.63;0.91)
**SBP (mmHg)**	No	ref		ref		ref	
	Yes	0.2	(−2.1;2.5)	0.0	(−2.3;2.3)	−0.3	(−2.7;2.0)
**DBP (mmHg)**	No	ref		ref		ref	
	Yes	−1.1	(−2.3;0.2)	−0.9	(−2.1;0.3)	−1.0	(−2.1;0.3)

Values are presented as mean difference in number of antihypertensive drugs or difference in blood pressure (with 95% Confidence Interval).

^1^ Adjusted for age and sex.

^2^ Adjusted for age, sex, BMI, Diabetes Mellitus, Cardiovascular disease, eGFR.

Abbreviations: ESA = Erythropoiesis-Stimulating Agent, SBP = Systolic Blood Pressure, DBP = Diastolic Blood pressure, ref = Reference, BMI = Body Mass Index, eGFR = estimated Glomerular Filtration Rate.

### BP during Pre-dialysis Care

There was no relevant difference in measured SBP and DBP during pre-dialysis care in patients with and without ESA treatment. In ESA treated patients SBP was just 0.2 mmHg higher (95% CI −2.1;2.5) and DBP 1.1 mmHg lower (95% CI −2.3;0.2) than in patients without ESA. Adjustment for age and sex and further adjustment for BMI, diabetes mellitus, cardiovascular disease and eGFR affected estimates just minimally. In patients with ESA treatment however, patients treated with higher ESA dose seemed to have on average higher BP, although confidence intervals are wide (Table 4). Patients treated with an ESA dose >6000 units/week had a 3.7 mmHg (95% CI −1.6;9.0) higher SBP than patients treated with the lowest ESA dose category. Differences in DBP were very small with 1.1 mmHg (95% CI −1.7;3.8) higher DBP in patients the highest ESA dose as compared to the lowest. Results did not materially change with further adjustment for hemoglobin and there was no significant interaction between ESA use or dose and time or eGFR. In addition, results were essentially the same when patients with a renal transplant were excluded.

**Table 4:** Difference in BP in patients with ESA according to dose.

	ESA (units/week)	Unadjusted		Adjusted^1^		Adjusted^2^	
**SBP** **(mmHg)**	10–2000	ref		ref		ref	
	2001–4000	1.0	(−3.3;5.3)	1.2	(−3.1;5.4)	0.7	(−3.6;4.8)
	4001–6000	1.3	(−4.2;6.8)	1.7	(−3.8;7.1)	1.2	(−4.3;6.6)
	>6000	5.2	(−0.1;10.4)	5.0	(−0.2;10.2)	3.7	(−1.6;9.0)
**DBP** **(mmHg)**	10–2000	ref		ref		ref	
	2001–4000	0.1	(−2.1;2.3)	0.0	(−2.2;2.2)	0.2	(−2.0;2.4)
	4001–6000	0.5	(−2.4;3.3)	0.4	(−2.4;3.3)	0.7	(−2.1;3.6)
	>6000	0.5	(−2.3;3.2)	0.5	(−2.2;3.3)	1.1	(−1.7;3.8)

Values are presented as mean difference in blood pressure (with 95% Confidence Interval).

^1^ Adjusted for age and sex.

^2^ Adjusted for age, sex, BMI, Diabetes Mellitus, Cardiovascular disease, eGFR.

Abbreviations: ESA = Erythropoiesis-Stimulating Agent, SBP = Systolic Blood Pressure, DBP = Diastolic Blood pressure, ref = Reference, BMI = Body Mass Index, eGFR = estimated Glomerular Filtration Rate.

## Discussion

This study showed an increased amount of antihypertensive drugs in pre-dialysis patients with ESA as compared to patients without ESA. This is in line with meta-analyses performed on trials that compared patients with and without ESA, which identified a 26% to twofold increased risk for an increase in antihypertensive agents in patients treated with ESA [33], [34]. Meta-analyses of anemia correction trials in CKD patients also reported a higher risk for hypertension or hypertension adverse events (including an escalation of the antihypertensive regimen) among patients treated to higher hemoglobin targets, with on average higher ESA doses [35], [36]. Whereas our study did detect a difference in antihypertensive medication, no relevant differences were found in routinely measured BP between patients with and without ESA. This confirms that in clinical practice actual BP can remain stable during ESA treatment, with adjustments in antihypertensive treatment [19], [20], [22], [37]. Thus in our study population, physicians were able to control BP in ESA treated patients to the same level as patients without ESA treatment. It should be noted that in both groups BP targets were hard to reach in clinical practice. In just 20.3% of patients BP was optimally controlled at the start of pre-dialysis care, of which 26.7% met criteria for SBP and 50.6% for DBP. This is in line with other reports in CKD patients [2], [23]–[25] and in pre-dialysis patients specifically [4].

The rise in BP during the use of ESA is multifactorial and includes (among others) a direct effect on endothelial function [38]. ESA increases the expression of endothelin-1 in resistance subcutaneous arteries from chronic kidney disease patients [39]. The rise in the patients’ BP could be attributed to the vasoconstrictive effects of endothelin-1 [40]. ESA treated endothelial cells also show a dose-dependent decrease in the vasodilating nitric oxide [41]. In addition, elevation of BP usually coincides with the rise in hematocrit and erythrocyte mass and thereby the increase in blood viscosity. It is, however, also reported that the increase in BP is independent of hematocrit [42].

ESA induced hypertension has already been reported in 10–32% of hemodialysis patients at the introduction of ESA into clinical practice [17]–[19]. Aside from the increased antihypertensive drug use in patients with ESA, the BP raising effect of ESA is also suggested in ESA treated patients in our study. Although confidence intervals are wide, a trend towards higher BP with high ESA dose is indicated. Other studies also reported higher incidences of hypertension with increasing ESA doses [43] or a dose dependent effect of ESA on DBP in hemodialysis patients [44], [45]. In pre-dialysis patients a secondary analysis of CHOIR reported an association between increases in ESA dose and increases in mainly DBP [21]. In this last report, however, increases in DBP were not associated with the composite endpoint of death, congestive heart failure hospitalization, stroke and myocardial infarction.

The debate about the safety of ESAs was started after the publication of several anemia correction trials in CKD patients in which high hemoglobin targets and therefore higher ESA doses were associated with increased mortality or cardiovascular events [10]–[12], [46]. The hypothesized mechanism for these adverse effects includes the elevation of BP by ESA, besides changes in endothelial function and effects of ESA on platelets and the coagulation system [16], [47].

Our results also support the hypothesis that ESA treatment affects BP. However, it seems less likely that routinely measured BP could explain a possible increase in cardiovascular events and mortality in our population as the increase in BP seems to be controlled for by antihypertensive medication. BP variability could still play a role, since it is associated with mortality [48] and a peak BP shortly after ESA administration is probably not captured in our data.

Some limitations mainly because of the observational nature of this study should be addressed. First, BP measurements reflect measurements as routinely taken in clinical practice under the influence of medication, both ESA and antihypertensive drugs and including a possible white-coat effect. A previous study has reported unchanged office BP after ESA treatment, but did detect an increased level of BP measured at home in the morning [49]. Second, data on the interval between erythropoietin injection or start of ESA treatment and BP measurement was lacking. ESA has been reported to exert both an immediate effect as a long-term effect on BP [38], [45], [50]. Since our analyses are cross-sectional in nature, a causal relation between ESA and BP is hard to establish. Third, different types of ESA were analyzed together. Previously no difference in BP response between darbepoetin or epoetin in pre-dialysis patients was reported [36], [51], and although numbers are small, a stratified analysis according to type of ESA in our data showed the same trend. Fourth, although the increase in antihypertensive medication was shown to the best of our abilities by an increase in antihypertensive drug types and confirmed by an increase in total standardized daily dose, the exact amount of drug use remains difficult to compare between groups. Adding up percentages of defined daily doses of different antihypertensive drugs is a rough estimate, since for some antihypertensive drug classes the dose in clinical practice can easily exceed the defined daily dose, while for others it rarely does. Last, in our observational study patients were not randomly allocated to a certain ESA treatment. The observational nature of our study therefore requires careful adjustment for confounding, which we executed the best we could, but residual confounding cannot be excluded.

In summary, in this prospective cohort of pre-dialysis patients we showed that ESA treated patients received more antihypertensive agents and no difference in routinely measured BP between patients with and without ESA was found. This means that the hypertensive effect of ESA, as also illustrated by the trend towards higher BP with high ESA dose, can be controlled to the same extent as patients without ESA in clinical practice. It seems therefore questionable from our results that this effect could contribute to the increased cardiovascular risk associated with ESA use in pre-dialysis patients. Trials evaluating the ESA-induced risk of cardiovascular events should assess whether this risk was mediated by elevated BP and the influence of other mechanisms should be investigated.

## Supporting Information

File S1 **Supporting Information File S1 contains additional information on the ethics statement including a list of the medical ethics committees or institutional review boards of all participating centers.**
(DOC)
