# Supplementary material for: Effect of Erythropoiesis-Stimulating Agents on Blood Pressure in Pre-Dialysis Patients
Source: PLoS One. 2013 Dec 31;8(12):e84848. doi: 10.1371/journal.pone.0084848 (PMC3877353; doi:10.1371/journal.pone.0084848)
Supplement: File S1 — Supporting Information File S1 contains additional information on the ethics statement including a list of the medical ethics committees or institutional review boards of all participating centers. (DOC) [file pone.0084848.s001.doc]

**Supporting Information File S1: Ethics Statement**

The PREPARE-2 study was reviewed and approved by the medical ethics committee of the Leiden University Medical Center. The medical ethics committee or institutional review board (as appropriate) of all participating centers additionally reviewed and approved the local feasibility of the study, according to the Central Committee on Research Involving Human Subjects (CCMO) External Review Directive^*^. These centers are:

- Amsterdam, Academic Medical Center
- Amsterdam, Sint Lucas-Andreas Hospital
- Amsterdam, VU Medical Center
- Apeldoorn, Gelre Hospitals
- Breda, Amphia Hospital
- Delft, Reinier de Graaf
- Den Bosch, Jeroen Bosch Hospital
- The Hague, Medical Center Haaglanden
- Ede, Hospital Gelderse Vallei
- Eindhoven, Catharina Hospital
- Emmen, Scheper Hospital (approval also included Beilen, Dialysis Clinic North)
- Goes, Admiraal de Ruyter Hospital
- Gouda, Groene Hart Hospital
- Groningen, University Medical Center Groningen
- Haarlem, Kennemer Gasthuis
- Leeuwarden, Medical Center Leeuwarden
- Leiderdorp, Rijnland Hospital
- Leiden, Leiden University Medical Center
- Roermond, Laurentius Hospital
- Roosendaal, Franciscus Hospital
- Rotterdam, Franciscus Gasthuis
- Veldhoven, Máxima Medical Center
- Zaandam, Zaans Medical Center
- Zwolle, Isala Clinics

^*^ [http://www.ccmo-online.nl/hipe/uploads/downloads_catm/CCMO%20External%20Review%20 Directive%202012%20dated%2014-12-2011.pdf](http://www.ccmo-online.nl/hipe/uploads/downloads_catm/CCMO External Review  Directive 2012 dated 14-12-2011.pdf )
